# Supplementary material for: A New Method for Conditional Gene-Based Analysis Effectively Accounts for the Regional Polygenic Background
Source: Genes (Basel). 2024 Sep 7;15(9):1174. doi: 10.3390/genes15091174 (PMC11431718; doi:10.3390/genes15091174)
Supplement: Supplementary file 1 [file genes-15-01174-s001.zip › Supplementary Materials_Svishcheva_.pdf]

Supplementary Materials

A new method for conditional gene-based analysis effectively accounts for the regional polygenic background

Svishcheva G.R., Belonogova N.M., Kirichenko A.V., Tsepilov Y.A., Axenovich T.I.

Supplementary Notes

S1. The maximum-likelihood estimator (MLE)

In order to estimate the value of τ, a -2 × natural logarithm (ln) of likelihood function is constructed for the vector z_r:

$$-2\, ln\, Lh \sim \ln|\tau U_r U_r + U_r| + z_r^T (\tau U_r U_r + U_r)^{-1} z_r.$$

Using simple transformations, we obtained:

$$-2\, ln\, Lh \sim \ln|U_r| + \ln|\tau U_r + I| + z_r^T U_r^{-\frac{1}{2}} (\tau U_r + I)^{-1} U_r^{-\frac{1}{2}} z_r.$$

Since the summand $ln|U_r|$ is independent of τ, it can be omitted:

$$-2\, ln\, Lh \sim \ln|\tau U_r + I| + z_r^T U_r^{-\frac{1}{2}} (\tau U_r + I)^{-1} U_r^{-\frac{1}{2}} z_r.$$

To reduce the running time for MLE, some analytical transformations are first carried out. Using the properties of matrix form of A*x+I (from Linear algebra), we obtained expressions for summands of $(-2\, ln\, Lh)$:

$$\ln|\tau U_r + I| = \sum_{i=1}^{k} \ln(\tau \lambda_i + 1)$$

and

$$(\tau U_r + I)^{-1} = V \left\{\frac{1}{\tau \lambda_i + 1}\right\} V^T.$$

Here λ_i is an (m_r×1) vector of eigenvalues for the matrix U_r; V is an (m×m) matrix of eigenvectors for the matrix U_r; curly brackets define the diagonalization of vector and k

is the rank of matrix. The k parameter restricts the vector of eigenvalues to be analysed. It is typical for LD matrices to exhibit multicollinearity; therefore, they are approximated by semi-defined low-rank matrices, for which a pseudo-inverse matrix is then sought.

After substituting these expressions, we obtained:

$$-2\, ln\, Lh \sim \sum_{i=1}^{k} \ln(\tau\lambda_i + 1) + \sum_{i=1}^{k} \left\{ \frac{\tilde{z}_r^2}{\tau\lambda_i + 1} \right\},$$

where $\tilde{z} = z_r^T U_r^{-\frac{1}{2}}$.

Next, the optimisation problem of identifying the optimal value of τ is solved.

S2. TauCOR property

TauCOR has a property depending on the selected GBA test. When kernel-based tests such as Burden, SKAT, or SKAT-O are used in a conditional GBA analysis, the p-values are guaranteed to be greater than or equal to the p-values of the initial GBA analysis.

This follows from the distribution of the kernel-based score test statistics that can be written in general form for Burden, SKAT or SKAT-O:

$$Q = z_g^T R_{\theta} z_g,$$

where $R\theta = {}_{\theta}\mathbf{11}^T + (1 - \theta) I_{m_g}$ is the correlation matrix between intragenic SNP effects, and θ is the parameter regulating the selection of the kernel-based test (Burden when θ is fixed at 1; SKAT when θ is fixed at 0, and SKAT-O when θ is not fixed and estimated in the range [0, 1]) (Lee *et al.* 2012). In accordance with Exp.(8) from the main text, the test statistic Q is distributed as a weighted sum of $\chi^2_{df=1}$-distributions with weights equal to the eigenvalues of the corrected LD matrix, $(\tau\, U_{gr} U_{gr}^T + U_g) R_{\theta}$. Since the matrices U_g and $\tau U_{gr} U_{gr}^T$ are positively semi-definite, the eigenvalues of $(\tau\, U_{gr} U_{gr}^T + U_g) R_{\theta}$ increase as τ increases. Consequently, the p-value of the test statistic also increases.

S3. Gene-level LDSC regression

Bulik et al. (Bulik-Sullivan *et al.* 2015) have developed the LDSC regression model for a single SNP. This model accounts for polygenic background around a single SNP, designated as SNP i:

$$Cov(z_{g_i}|z_r) = l_i \frac{nh_r^2}{m_r} + 1 + na. \quad \text{(S1)}$$

Here n is the sample size; m_r is the number of SNPs in a region of interest; h_r^2 is the regional (local) heritability; a measures the contribution of confounding biases, such as cryptic relatedness and population stratification; l_i is the LD score between the i-th SNP and all other SNPs from the surrounding region, LD score is calculated as $\ell_i \coloneqq \Sigma_j u_{ij}^2$, where u_{ij} is the correlation coefficient between the i-th and k-th SNPs.

It is assumed that the summary statistics have already been adjusted for cryptographic relatedness and population stratification, and thus that $a = 0$. In this case, Formula (S1) can be simplified as

$$Cov(z_{g_i}|z_r) = \sum_{j=1}^{m_r} u_{ij}^2 \frac{nh_r^2}{m_r} + 1. \quad \text{(S2)}$$

It is important to note that Formula (S2) is a particular case of Formula (7) described in the main text. Formula (7) was derived for the covariance matrix of SNPs within a gene, with the aim of accounting for regional polygenic influence under the null hypothesis of no gene association:

$$Cov(z_g|z_r) = U_{gr}U_{gr}^T \frac{nh_r^2}{m_r} + U_g.$$

Consequently, the aforementioned formula represents an extension of the LDSC regression, which has been previously constructed at the single-SNP level, to the gene level.

S4. On the conversion from a fixed-effects model to a random effects model

Formula (8) that describes the parameters of the conditional distribution of z-statistics of intragenic SNPs can also be obtained from the corresponding formula proposed in (Pasaniuc *et al.* 2014), where the extragenic SNP effects were assumed as fixed. In accordance with our notations, the conditional distribution of z-statistics obtained in (Pasaniuc *et al.* 2014) can be written:

$$f(z_g|z_r) \Leftarrow \begin{cases} E(z_g|z_r) = \boxed{U_{gr}U_r^{-1}z_r} \\ Cov(z_g|z_r) = U_g - \boxed{U_{gr}U_r^{-1}U_{gr}^T} \end{cases} \quad \text{(S3)}$$

As can be seen from Formula (S3), in the conditional GBA analysis based on fixed-effects model, the biases are observed in both the mean vector and the covariance matrix (these biases are highlighted by the boxes in Formula (S3)).

As follows from linear algebra, without loss of generality, we can remove the bias in the mean vector, transforming it into a bias in the covariance matrix:

$$f(z_g|z_r) \Leftarrow \begin{cases} E(z_g|z_r) = 0 \\ Cov(z_g|z_r) = U_g - \boxed{U_{gr}U_r^{-1}U_{gr}^T} + \boxed{U_{gr}U_r^{-1}z_r z_r^T U_r^{-1}U_{gr}^T} \end{cases}$$

We can now consider the extra-genic SNP effects not as fixed but as random and distributed as $f(z_r) \sim N(\mathbf{0}, U_r + \tau U_r^2)$) (see Exp.(4)). This leads to the following formula:

$$f(z_g|z_r) \Leftarrow \begin{cases} E(z_g|z_r) = 0 \\ Cov(z_g|z_r) = U_g - \boxed{U_{gr}U_r^{-1}U_{gr}^T} + \boxed{U_{gr}U_r^{-1}E(z_r z_r^T)U_r^{-1}U_{gr}^T} \end{cases}$$

Since $E(z_r z_r^T) = U_r + \tau U_r^2$, we obtained

$$f(z_g|z_r) \Leftarrow \begin{cases} E(z_g|z_r) = 0 \\ Cov(z_g|z_r) = U_g - \boxed{U_{gr}U_r^{-1}U_{gr}^T} + \boxed{U_{gr}U_r^{-1}(U_r + \tau U_r^2)U_r^{-1}U_{gr}^T} \end{cases}$$

The two matrices in boxes were then added together:

$$f(z_g|z_r) \Leftarrow \begin{cases} E(z_g|z_r) = 0 \\ Cov(z_g|z_r) = U_g + \boxed{U_{gr}U_r^{-1}(-U_r + U_r + \tau U_r^2)U_r^{-1}U_{gr}^T} \end{cases}$$

This formula can be simplified to become equivalent to our Formula (8).

$$f(z_g|z_r) \Leftarrow \begin{cases} E(z_g|z_r) = 0 \\ Cov(z_g|z_r) = U_g + \boxed{\tau U_{gr}U_{gr}^T} \end{cases} .$$

Supplementary Tables

Table S1. Parameters and inputs for the simulation model that directly simulates summary statistics

Model parameters	Description	Values
ρ	The proportion of causal SNPs in gene among all causal SNPs	0 or 1
h_{GW}^2	Genome-wide heritability	0.3, 0.5 or 0.7
K	The total number of causal SNPs	10
**Initial data**	**Description**	**Values**
U_r	LD matrix for setR	real
U_g	LD matrix for setG	real
U_{rg}	LD matrix between setR and setG	real
m_g	The number of SNPs in gene	real
m_r	The number of SNPs in surrounding region	real
M	The number of SNPs in the genome	8 000 000
n	Sample size	380 506

Table S2. The specificity and sensitivity of the initial and conditional GBA analyses in the simulation study

Scenarios			The number of runs		Specificity		
**GBA test**	ρ	h_{GW}^2	N_{tot} *	N_{an}	**Initial GBA**	COJO+GBA	TauCOR+GBA
	0	0.3	129111	3716	0.971	0.12	0.068
**PCA**	0	0.5	87568	5561	0.936	0.099	0.081
	0	0.7	66352	6319	0.904	0.103	0.101
	0	0.3	612000	5159	0.992	0.564	0.463
**BT**	0	0.5	580000	11440	0.980	0.543	0.493
	0	0.7	551726	17456	0.968	0.532	0.518
	0	0.3	216000	6635	0.969	0.089	0.525
**SKAT**	0	0.5	216000	14571	0.932	0.061	0.515
	0	0.7	216000	22408	0.894	0.044	0.502
**Scenarios**			**The number of runs**		**Sensitivity**		
	1	0.3	14237	8149	0.626	0.934	0.996
**PCA**	1	0.5	13048	8113	0.801	0.949	0.998
	1	0.7	51385	27962	0.912	0.976	0.996
	1	0.3	108000	26401	0.262	0.682	0.901
**BT**	1	0.5	108000	34897	0.373	0.678	0.882
	1	0.7	108000	38709	0.444	0.674	0.867
	1	0.3	108000	61163	0.616	0.968	0.929
**SKAT**	1	0.5	108000	68756	0.784	0.976	0.934
	1	0.7	107266	65783	0.864	0.982	0.954

* N_{tot}: the total number of runs; N_{an}: the number of runs permitted for conditional analysis

Table S3. The accuracy of the initial and the conditional GBA analyses in the simulation study

Scenarios		Accuracy		
**GBA test**	h_{GW}^2	Initial GBA	COJO+GBA	TauCOR+GBA
**PCA**	03	0.934	0.742	0.768
	05	0.917	0.613	0.634
	07	0.898	0.696	0.712
**BT**	03	0.887	0.662	0.825
	05	0.893	0.641	0.779
	07	0.894	0.624	0.748
**SKAT**	03	0.851	0.882	0.887
	05	0.883	0.816	0.859
	07	0.884	0.744	0.829

Table S4. The standard deviations calculated for the differences between the *log₁₀*(*p*)-values of the initial and conditional GBA analyses in the simulation study

GBA test	ρ	h_{GW}^2	sd(COJO)*	sd(TauCOR)
**PCA**	0	0.3	16.94	1.24
	0	0.5	19.03	1.88
	0	0.7	21.01	2.50
	1	0.3	11.36	1.23
	1	0.5	14.99	1.73
	1	0.7	13.99	2.19
**BT**	0	0.3	5.23	1.55
	0	0.5	5.92	2.10
	0	0.7	6.33	2.50
	1	0.3	6.07	1.76
	1	0.5	6.63	2.23
	1	0.7	7.03	2.62
**SKAT**	0	0.3	13.45	1.70
	0	0.5	14.36	2.31
	0	0.7	15.17	2.87
	1	0.3	7.98	1.96
	1	0.5	8.49	2.53
	1	0.7	8.76	2.97

* sd(*X*): the standard deviation calculated for the differences between the $log_{10}(p)$-values of the initial GBA analysis and conditional *X*-based GBA analysis, where *X* denotes either TauCOR or COJO.

Supplementary Figures

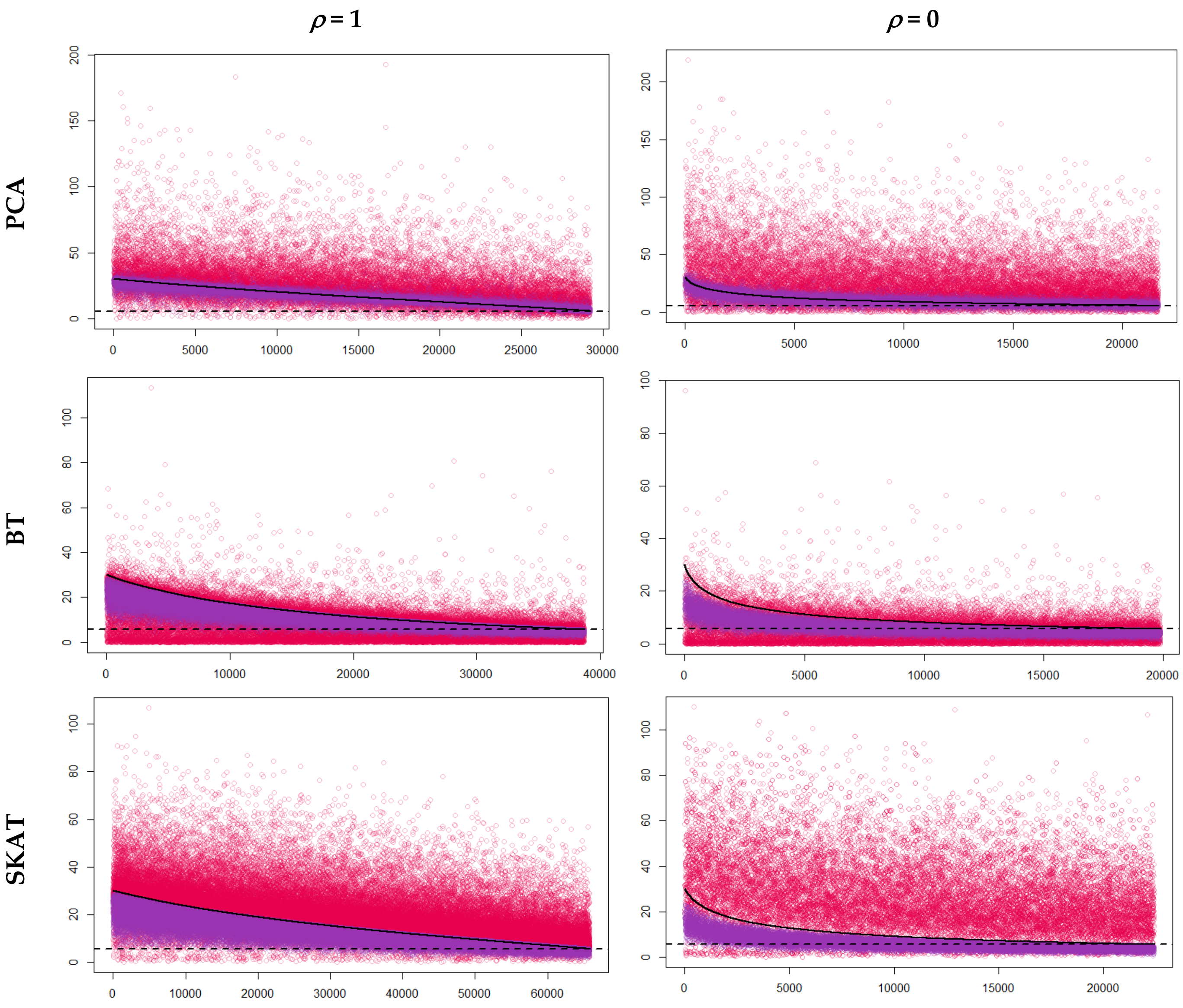

Figure S1. The -log$_{10}$(p) values of the conditional GBA analyses on the simulated summary data for two scenarios, where $\rho = 0$ and $\rho = 1$ under $h_{GW}^2 = 0.7$.

On each plot, the -log$_{10}$(p) values were calculated in the initial, COJO- and TauCOR-based GBA analysis. The initial GBA p-values are presented in ascending order and are marked by the black continuous line. The red dots represent COJO, while the blue dots represent TauCOR. The dotted line indicates the GBA threshold equal to $-log_{10}(2.5{\times}10^{-6})$.

References

Bulik-Sullivan B.K., Loh P.R., Finucane H.K., Ripke S., Yang J., Schizophrenia Working Group of the Psychiatric Genomics C., Patterson N., Daly M.J., Price A.L. & Neale B.M. (2015) LD Score regression distinguishes confounding from polygenicity in genome-wide association studies. *Nat Genet* **47**, 291-5.

Lee S., Wu M.C. & Lin X. (2012) Optimal tests for rare variant effects in sequencing association studies. *Biostatistics* **13**, 762-75.

Pasaniuc B., Zaitlen N., Shi H., Bhatia G., Gusev A., Pickrell J., Hirschhorn J., Strachan D.P., Patterson N. & Price A.L. (2014) Fast and accurate imputation of summary statistics enhances evidence of functional enrichment. *Bioinformatics* **30**, 2906-14.
